# The Restorative Effect of Red Guava (*Psidium guajava* L.) Fruit Extract on Pulmonary Tissue of Rats (*Rattus norvegicus*) Exposed to Cigarette Smoke

**DOI:** 10.1155/2021/9931001

**Published:** 2021-06-01

**Authors:** Dewa Ketut Meles, Imam Mustofa, Wurlina Wurlina, Suherni Susilowati, Suzanita Utama, Niluh Suwasanti, Desak Ketut Sekar Cempaka Putri

**Affiliations:** ^1^Faculty of Veterinary Medicine Airlangga University, Kampus C, Mulyorejo, Surabaya 601155, East Java, Indonesia; ^2^Faculty of Medicine of Widya Mandala Catholic University, Jalan Raya Kalisari Selatan, Kampus Pakuwon City, Surabaya 60112, East Java, Indonesia; ^3^Faculty of Medicine Universitas Airlangga, General Hospital Dr. Soetomo, Jl. Major General Professor Dr. Moestopo No. 6–8, Surabaya 60286, East Java, Indonesia

## Abstract

Since the damage to alveolar tissue due to cigarette smoke exposure (CSE) is lipid peroxidation, antioxidant treatment is needed. The red guava (*Psidium guajava* L.) fruit contains antioxidants derived from quercetin, lycopene, and vitamin C. This study aimed to determine the effect of red guava fruit extract (RGFE) on the alveolar tissue of rats exposed to cigarette smoke. The 25 rats (*Rattus norvegicus*) were divided into five groups. The control and T0 groups were only administered placebo, while T1, T2, and T3 groups were orally administered RGFE of 18.9, 37.8, and 56.7 mg/kg body weight daily for 44 days. The CSE dose of 20 suctions daily was conducted on T0, T1, T2, and T3 groups on days 15–44. On day 45, all rats were sacrificed for serum collection and histopathological lung slides with eosin-nigrosin staining. The result showed that CSE caused an increase (*p* < 0.05) in malondialdehyde (MDA) levels, cell death, apoptosis, and necrosis percentages, congestion and thickening of alveolar septum tissue, and reduction in the alveolar diameter and alveolar number. Administration of RGFE suppressed those effects, and the highest dose of RGFE (T3) restored (*p* > 0.05) MDA levels, percentage of apoptotic and necrosis, alveolar septal thickening, and alveolar diameter. However, the percentages of cell death, alveolar congestion, and the alveolar number were still worse (*p* < 0.05) than in normal rats. It could be concluded that RGFE has proved relief and restoration of the alveolar tissue of rats exposed to cigarette smoke.

## 1. Introduction

The smoking epidemic is one of the biggest public health threats the world has ever faced. It causes more than 8 million people to die a year worldwide due to obstructive pulmonary disease and lung cancer [1]. Currently, about 19% of adults around the world smoke tobacco [2]. Secondhand smoke increases the risk of cancer, particularly lung cancer [3]. Also, smokers are vulnerable to respiratory viruses. Smoking can upregulate the angiotensin-converting enzyme-2 (ACE2) receptor, the known receptor for the severe acute respiratory syndrome (SARS) coronavirus 2 (SARS-CoV-2). ACE2 could be an adhesion molecule for SARS-CoV-2, causing COVID-19 [4].

The gas mixture of cigarette smoking contains about 4,500 components, such as carbon monoxide, nicotine, oxidants, delicate particulate matter, and aldehydes. These components were the principal factors of the pathogenesis and progression of pulmonary disease [5]. Efficacious nonpharmacologic interventions, such as self-help programs, counseling, cognitive-behavioral therapy, and healthcare provider interventions, and pharmacologic agents such as nicotine replacement (gum, lozenge, spray, etc.), bupropion, and varenicline, are needed to quit smoking cigarettes [6]. Therefore, it is necessary to explore how antioxidants can resolve pulmonary tissue damage due to cigarette smoke in a rat model for good health and well-being. The mature red guava showed the best antioxidant activity [7]. Major phytochemical constituents isolated from the red-colored pulp flesh of *Psidium guajava* are quercetin (a flavonoid), lycopene (a carotenoid) [8], and vitamin C [9]. Vitamin C (ascorbic acid) levels were higher in red guava than the white guava [10, 11]. The extract of red guava demonstrated a promising effect against oxidative stress [12]. Therefore, the study aimed to determine the effect of red guava fruit extract (RGFE) in restoring the alveolar tissue based on serum levels of malondialdehyde (MDA), cell death, apoptosis, necrosis, alveolar congestion, the thickness of alveolar septum, alveolar diameter, and the alveolar number of rats exposed to cigarette smoke (CS).

## 2. Materials and Methods

### 2.1. Ethical Approval

This study's procedure was approved by the Animal Care and Use Committee, Airlangga University, Surabaya, Indonesia, No. 324/HRECC.FODM/VI/2020.

### 2.2. Extraction of Guava Fruit

Extraction of guava fruit was conducted based on the modification of the earlier report [13]. 1.5 kilograms of red guava fruit were dried by aeration at room temperature, and then, 50 grams of the dried powder underwent Soxhlet extraction with 600 ml of *n*-hexane for 6 hours. The residue was subjected to reflux with 400 ml of dichloromethane methanol (2 : 1) for 30 minutes, three times. The filtrate was concentrated to form a crude extract and then subjected to reflux with 200 ml of dichloromethane ethyl acetate (1 : 1) for 30 minutes seven times. The precipitate was dissolved in dichloromethane methanol (2 : 1) 100 ml and concentrated to 40 ml through evaporation and then dropped into 100 ml of acetone. The sediment was placed in an oven set to 40°C to obtain RGFE [14].

### 2.3. RGFE Dosage

The main effect of RGFE was the antioxidative activity to annihilate lipid peroxidation. CS induces lipid peroxidation of cell membranes followed by increases in MDA levels. Our preliminary study revealed that a dose of 18.9 g/kg body weight was the minimum dose that significantly decreases the MDA levels of rats exposed to CS [15]. Therefore, the RGFE doses in this study were designed in multiples of 18.9 g/kg body weight to determine the effective dose for restoring the pulmonary tissue of rats (*Rattus norvegicus*) exposed to cigarette smoke.

### 2.4. Treatment of Experimental Animals

The 25 healthy female Wistar rats (*Rattus norvegicus*) were randomly divided into five groups. The control group (CG) and T0 group were only administered sodium carboxymethylcellulose (Na CMC) at a concentration of 0.5%. T1, T2, and T3 groups were administered with RGFE at a dose of 18.9, 37.8, and 56.7 mg/kg body weight/day, respectively. Administration of Na CMC and RGFE was conducted in 0.4 ml orally before feeding daily for 44 days. The T0, T1, T2, and T3 groups were exposed to electric cigarette smoke with a pure tobacco dose of 20 suctions daily on days 15–44. On day 45, all rats were sacrificed to examine serum MDA levels and the histopathological slide with eosin-nigrosin staining. Observations were made on a 400x magnification light microscope (Nikon E200) equipped with Optilab Viewer Software Version 2.2.

The group's parameters were evaluated using variance (ANOVA) and followed by Tukey's Honestly Significant Difference test. Statistical analysis was conducted to a 95% level of significance using Statistical Product and Service Solutions Version 23.

### 2.5. Examination of Pulmonary Alveolar

The measurement of the alveolar congestion and thickening of the alveolar septum was performed using a scoring system on five fields of view and then averaged. The scoring system was based on the following criteria: no changes of alveolar congestion/thickening of the alveolar septum, less than 10%, 11–25%, 26–50%, and 51–75%, and more than 75% alveolar congestion/thickening of the alveolar septum of the visual field scored as 0, 1, 2, 3, 4, and 5, respectively.

### 2.6. Alveolar Cell Death Examination

Ethidiumbromide and acridine orange staining were used to examine pulmonary cell death (apoptosis and necrosis). The observation of apoptosis and necrosis cell death percentage was conducted under a fluorescent microscope (Olympus BX-51, Shinjuku City, Tokyo, Japan) at 400× magnification. Apoptotic cells were marked orange with bright spots, necrosis cells were orange without bright spots, and live cells were green.

### 2.7. MDA Level Measurements

The measurement of MDA concentration was conducted using the thiobarbituric acid method [16].

### 2.8. Alveolar Diameter

The alveolar diameter (*μ*m) was measured on five alveoli of each group at 400x magnification using a light microscope (Nikon E200) equipped with the Image Raster Software Version 3.7.

### 2.9. Alveolar Number

The alveolar number was measured on five views on each group at 100x magnification using a light microscope (Nikon E200).

### 2.10. Data Analysis

The data obtained were analyzed using one-way ANOVA followed by Tukey's Honestly Significant Difference test (SPSS Version 23) at a 95% level of significance.

## 3. Result

CSE in the model rats showed increased lung tissue damage, percentage of cell death, apoptosis, necrosis, alveolar congestion, and thickening of the alveolar septum and decreased alveolar diameter and alveolar count with increased MDA levels. RGFE administration resulted in the improvement of these damages.

### 3.1. Serum MDA Level and Alveolar Cell Death

**Figure 1:**
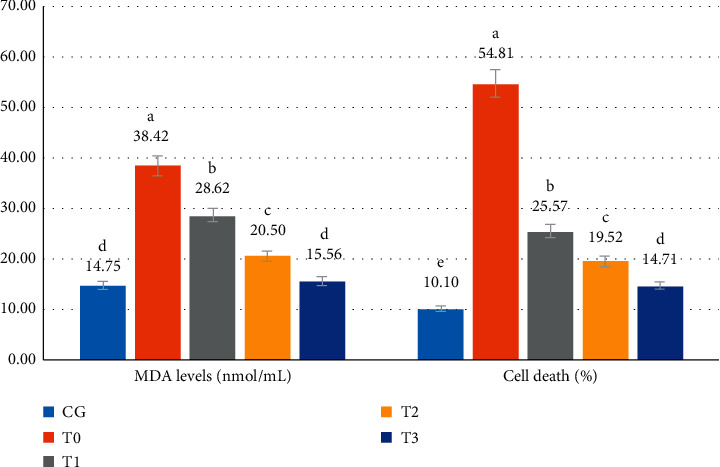
The serum MDA levels (ng/mL) and cell death (%) of rats exposed to cigarette smoke and administered RGFE. Different letters in a cluster were significantly different (*p* < 0.05). CG = rats administered 0.5 mL CMC Na 0.5%, T0 = rats administered CMC Na 0.5% and exposed to cigarette smoke, and T1, T2, and T3 = rats administered RGFE 18.9, 3.78, and 5.76 mg/kg BW/day and exposed to cigarette smoke.

CSE in the model rats caused an increase (*p* < 0.05) in MDA levels of 2.6-fold (from 14.75 ± 0.82 to 38.42 ± 1.87). RGFE administration reduced MDA levels so that they returned to the same level (*p* > 0.05) as normal rats (15.56 ± 1.47 in T3 rats vs. 14.75 ± 0.82 normal rats). CSE in model rats caused an increase (*p* < 0.05) in the percentage of cell death by 5.5-fold (10.10 ± 1.34% to 54.81 ± 4.12). RGFE administration decreased the percentage of cell death. However, at the highest dose of RGFE (T3), the percentage of cell death (14.71 ± 1.44%) was still higher (*p* < 0.05) than that in normal rats (Figure 1).

### 3.2. Cell Apoptosis and Necrosis

**Figure 2:**
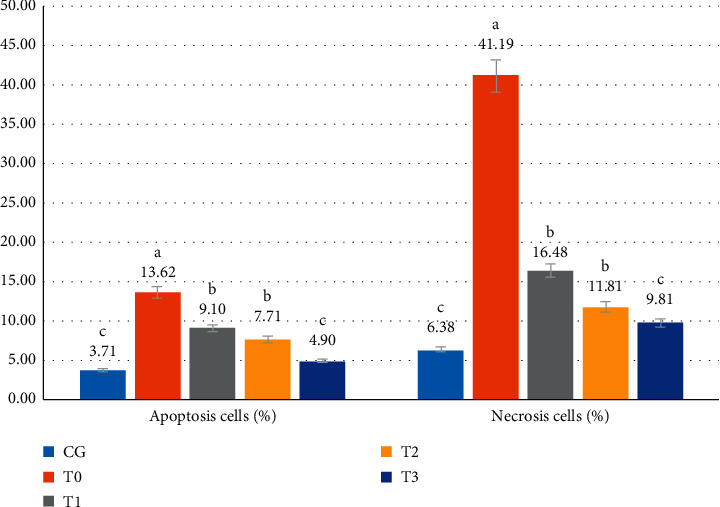
The apoptotic (%) and necrotic (%) cells on pulmonary alveolar exposed to cigarette smoke and administered RGFE. Different letters in a cluster were significantly different (*p* < 0.05). CG = rats administered 0.5 mL CMC Na 0.5%, T0 = rats administered CMC Na 0.5% and exposed to cigarette smoke, and T1, T2, and T3 = rats administered RGFE 18.9, 3.78, and 5.76 mg/kg BW/day and exposed to cigarette smoke.

**Figure 3:**
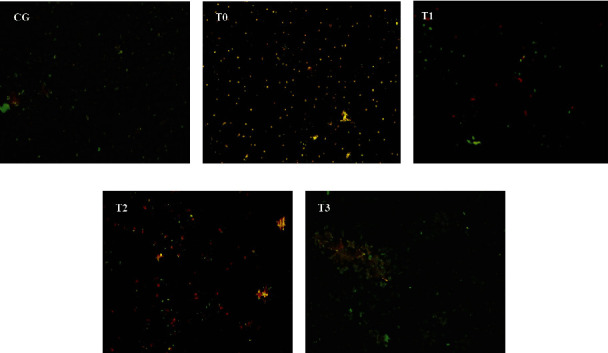
The pulmonary cells of the rat were administered RGFE and exposed to cigarette smoke. Ethidium bromide and acridine orange staining, magnification of 400X. Note: green color = alive pulmonary cells, orange color with yellow spots in the center of cells = apoptotic pulmonary cells, and brown color = necrotic of pulmonary cells. CG = rats administered 0.5 mL CMC Na 0.5%, T0 = rats administered CMC Na 0.5% and exposed to cigarette smoke, and T1, T2, and T3 = rats administered RGFE 18.9, 3.78, and 5.76 mg/kg BW/day and exposed to cigarette smoke. (a) CG. (b) T0. (c) T1. (d) T2. (e) T3.

CSE caused an increase (*p* < 0.05) in the percentage of apoptotic and necrotic cells by 3.7-and 6.5-fold. Apoptosis went from 3.71 ± 1.02% to 13.62 ± 1.14%, and necrosis went from 6.38 ± 0.64% to 41.19 ± 4.42%. It appears that mortality due to necrosis was much higher (*p* < 0.05) than cell death due to apoptosis. RGFE administration reduced the percentage of apoptotic and necrotic cells, such that it was not significantly different (*p* > 0.05) from that of normal rats (Figures 2 and 3).

### 3.3. Alveolar Congestion and Thickening of the Alveolar Septum

**Figure 4:**
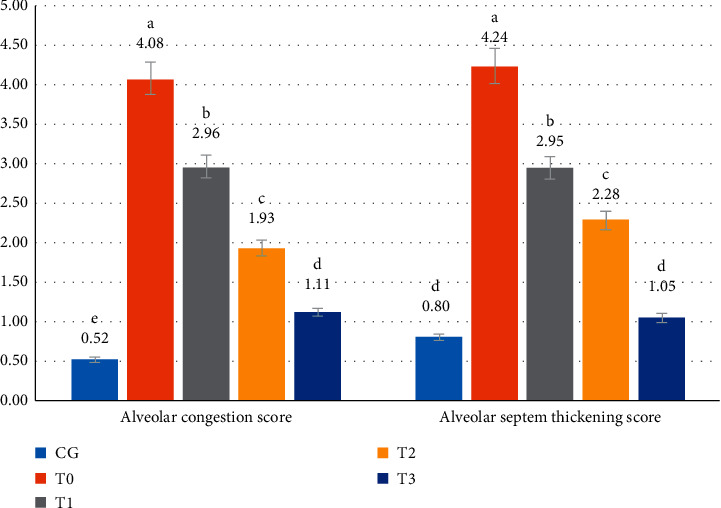
The score of the pulmonary alveolar congestion and alveolar septum thickening of rats exposed to cigarette smoke and administered RGFE. Different letters in a cluster were significantly different (*p* < 0.05). CG = rats administered 0.5 mL CMC Na 0.5%, T0 = rats administered CMC Na 0.5% and exposed to cigarette smoke, and T1, T2, and T3 = rats administered RGFE 18.9, 3.78, and 5.76 mg/kg BW/day and exposed to cigarette smoke.

**Figure 5:**
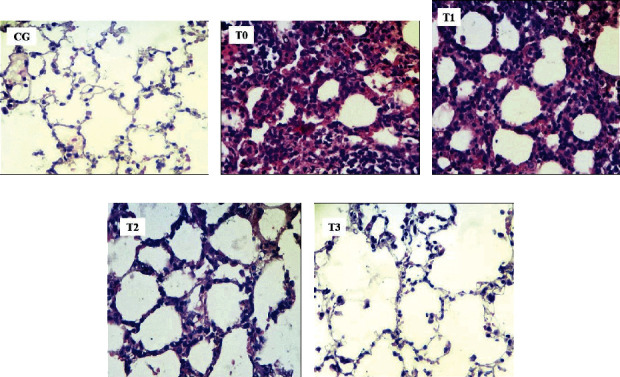
The pulmonary alveolar of rats administered RGFE and exposed to cigarette smoke. Hematoxylin and eosin staining, magnification of 400X. Note: arrow = alveolar congestion, CG = rats administered 0.5 mL CMC Na 0.5%, T0 = rats administered CMC Na 0.5% and exposed to cigarette smoke, and T1, T2, and T3 = rats administered RGFE 18.9, 3.78, and 5.76 mg/kg BW/day and exposed to cigarette smoke. (a) CG. (b) T0. (c) T1. (d) T2. (e) T3.

CSE in the model rats led to a higher congestion score (*p* < 0.05) of 7.8-fold (from a score of 0.52 ± 0.02 to a score of 4.08 ± 0.08). The administration of RGFE reduced the congestion score. However, up to the highest dose of RGFE (T3), the congestion score was still higher (*p* < 0.05), namely, 1.11 ± 0.03, compared to that of normal rats at 0.52 ± 0.02. CSE in the rat model caused the alveolar septal thickening (4.24 ± 0.03) to be higher (*p* < 0.05), at 5.3-fold, compared to the alveolar septum of normal rats (0.80 ± 0.26). Administration of RGFE reduced the thickness so that it was not significantly different (*p* > 0.05) compared to normal rats (Figures 4 and 5).

### 3.4. The Diameter Alveolar and Alveolar Count

**Figure 6:**
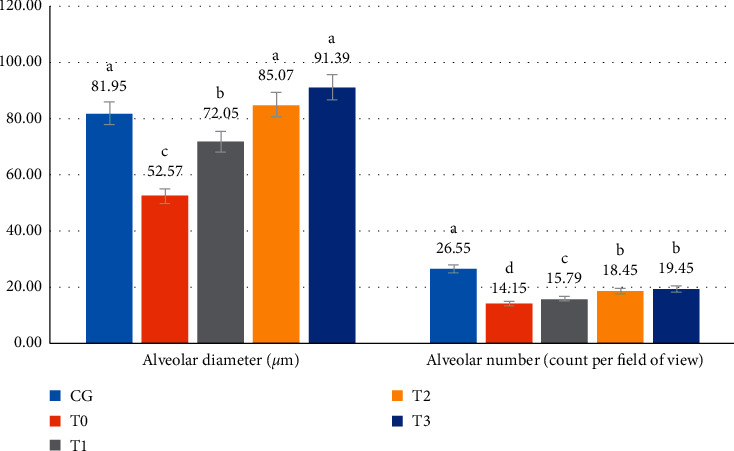
The diameter alveolar (*μ*m) and the number of alveolars (count per field of view) of rats exposed to cigarette smoke and administered RGFE. Different letters in a cluster were significantly different (*p* < 0.05). CG =  rats administered 0.5 mL CMC Na 0.5%, T0 = rats administered CMC Na 0.5% and exposed to cigarette smoke, and T1, T2, and T3 = rats administered RGFE 18.9, 3.78, and 5.76 mg/kg BW/day and exposed to cigarette smoke.

CSE in the rat model caused a 36% reduction (*p* < 0.05) in diameter (from 81.95 ± 4.55 to 52.57 ± 2.56). Administration of RGFE starting from T2 increased the diameter again so that it was not significantly different (*p* > 0.05) compared to normal rats. CSE in the rat model decreased (*p* < 0.05) the number of alveoli by 47% (from 26.55 ± 1.47 to 14.15 ± 0.69). Administration of RGFE starting from T2 increased (*p* < 0.05) the diameter but did not return it to the same (*p* < 0.05) diameter as normal rats. The highest dose (T3) shows the alveolar number at 19.45 ± 1.83, higher (*p* < 0.05) than in normal rats (Figures 5 and 6).

## 4. Discussion

The tar and gas which are the two main phases of cigarette smoke have been identified to be rich in oxidants that cause lipid peroxidation [17]. Red guava fruit contains antioxidants quercetin, L-ascorbic acid (vitamin C), and lycopene. Quercetin shows robust antioxidant activity by maintaining oxidative balance through its effect on glutathione (GSH), enzymatic activity, signal transduction pathways, and reactive oxygen species (ROS) [18]. L-ascorbic acid (vitamin C) protects cellular components against oxidative damage caused by ROS and acts as a radical scavenger for ROS and free radicals [19]. Meanwhile, lycopene enhances the production of endogenous antioxidant enzymes, such as glutathione peroxidase (Gpx), glutathione reductase (GR), and superoxide dismutase (SOD) [20]. Therefore, RGFE administration was expected to address alveolar cell damage due to CSE.

Smoking induces oxidative stress by producing ROS and weakening the antioxidant defense systems [21]. The activity of antioxidant enzymes (SOD, CAT, and GPX) was lower among active smokers [17]. In this study, CSE of rat models caused an increase in MDA levels of 2.6-fold as compared to normal rats. This result follows that of a previous report that smoking induces higher levels of MDA [21] and lowers vitamin C levels [22]. RGFE administration reduced MDA levels, such that, at the highest RGFE dose, the MDA levels returned to the same level as that of normal rats. Administration of quercetin markedly diminished the levels of oxidant molecules xanthine oxidase, nitric oxide, and MDA while increasing the levels of antioxidant enzymes [23]. Vitamin C elevated SOD and catalase levels, both of which are antioxidant enzymes [24]. Lycopene administration elevated GSH-Px activity, reduced ROS synthesis, reduced mitochondrial ROS and intracellular concentrations, and reduced MDA levels [25].

CSE in the rat model caused an increase in cell death by 5.5-fold compared to normal rats. CSE resulted in a dose-dependent increase in cell death due to the apoptosis-inducing factor, which is implicated in DNA damage and ROS-mediated cell death [26]. The nicotine component in CSE caused a dose-dependent increase in epithelial cell death [27]. CSE caused the destruction of the matrix, blood supply shortage, and epithelial cell death [28]. RGFE administration decreased the percentage of cell death. Lycopene suppressed the increase of ROS, the total number of alveolar leukocytes, lipid peroxidation, catalase activity, and DNA damage [29]. However, at the highest dose of RGFE, the percentage of cell death was still higher than that of normal rats, even though the MDA levels were similar to those of normal rats. The levels of MDA illustrate the levels of cell membrane damage [30]. Therefore, based on the MDA levels in the T3-group rat, cell membrane restoration has occurred. However, it seems the percentage of cell death that has not returned to the normal value could be due to insufficient doses, insufficient recovery time, or irreversible cell damage.

This study result followed previous reports that cigarette smoke causes an increase in the number of apoptotic cells, as follows. CSE increases tumor necrosis factor expression and a decrease in Il6 expression [31] and pulmonary endothelial cell apoptosis [28] because the nicotine caused a dose-dependent increase in caspase-3/7 activities [27]. Nicotine induces oxidative stress through the apoptotic pathway via activation of caspase-3 [32] due to mitochondrial damage [33]. The endothelial apoptosis contributes to the severity progression of chronic obstructive pulmonary disease [34]. Guava fruit contains polyphenol that is antiapoptotic in endothelial cells [35]. Quercetin protects DNA from free-radical damage, and the antioxidant activity of the quercetin-DNA complex is more potent than that of quercetin itself [36]. Vitamin C blocks ROS production and induction of caspase-3 activity [37]. Meanwhile, lycopene inhibits smoke-induced oxidative stress and promotes genome stability [38]. The antioxidant activity of lycopene can trap ROS, react with free radicals, protect cells against lipid peroxidation and oxidative DNA damage, and block mitochondrial DNA damage caused by ROS [39]. Taken together, the components of RGFE at the higher dose could restore the viability of alveolar cells from apoptotic death.

CSE induced a significant increase in the percentage of necrotic cells and induced the production of proinflammatory cytokines [40]. CSE disrupted iron homeostasis resulting in excessive oxidative stress. The labile iron accumulation and enhanced lipid peroxidation with concomitant nonapoptotic cell death occur during CSE [41]. Quercetin exerts anti-inflammatory effects on pulmonary damage by inducing cellular defense against oxidative stress through transcriptional upregulation of antioxidant proteins [42]. Vitamin C inhibited the TNF-*α*-induced activation of the mitogen-activated protein kinase (MAPKs) and nuclear factor-kappa B (NF-*κ*B) signaling [43]. The TNF is a regulator of the generation of ROS and reactive nitrogen species in balancing cell survival, apoptosis, and necroptosis [44].

In this study, the mortality due to necrosis was much higher than cell death due to apoptosis. In the CSE-only group rats, the percentage of cell death due to necrosis was three-fold higher than that of cell death due to apoptosis. It seems CSE can induce a switch from apoptotic to necrotic cell death in the airway epithelium. CSE induces neutrophil necrosis, leading to damage-associated molecular pattern (DAMP) release, which amplifies CS-induced inflammation by promoting airway epithelial proinflammatory responses [45]. The hydrophobic tar fraction's low concentrations induce DNA damage, resulting in a P53-dependent and BCL-XL-inhibitable death cascade. The release of apoptosis-inducing factors mediates apoptotic death signaling. Higher hydrophobic tar fraction concentrations also induce apoptotic-like signaling, but the signaling cascade is redirected to necrosis [46].

CSE in the rat models led to a higher congestion score that is 7.8-fold higher than that of normal rats. The imbalance between oxidants and antioxidants resulting from exposure to tobacco smoke led to oxidative stress, increased mucosal inflammation, and increased the expression of inflammatory cytokines [47]. The administration of RGFE reduced the congestion score. Treatment with anti-inflammatory medicinal herbs reduced leukocyte influx in the bronchoalveolar lavage, decreased the number of mast cells and macrophages in the lungs, and prevented pulmonary congestion in Wistar rats [48]. Vitamin C enhances cortisol production and potentiates the anti-inflammatory and endothelial cytoprotective effects of glucocorticoids [49].

CSE in the rat model caused the alveolar septal thickening score to be 5.3-fold higher compared to those of normal rats. Quercetin exerts antifibrogenic effects on pulmonary damage by inducing Nrf2 for cellular defense against oxidative stress and transcriptional regulation of antioxidant proteins [42]. Vitamin C reduced the recruitment of inflammatory cells, the secretion of IL-17, a cytokine involved in neutrophils migration, TGF-*β*, a profibrotic mediator, and collagen deposition [24]. Vitamin C also regulates alveolar fluid clearance, including cystic fibrosis transmembrane conductance regulator, aquaporin-5, the Na+/K+-ATPase pump, and epithelial sodium channel [49].

The CSE in this study's rat model caused a 36% reduction in alveolar diameter. To our knowledge, there is no report about the change of the alveolar diameter due to CSE. The decreases of the alveolar diameter may be due to the alveolar septal thickening. The tobacco smoke exposure results in increased permeability, mucus overproduction, impaired mucociliary clearance, increased release of proinflammatory cytokines and chemokines, and enhanced recruitment of macrophages and neutrophils [47]. This was followed by congested blood vessels and increased neutrophil infiltration, lung myeloperoxidase mRNA and protein increased in the nicotine-exposed rats [27], and surfactant secretion rat lungs [50]. Administration of RGFE starting from T2 increased the diameter again to that similar to a normal rat. Vitamin C is known to contribute to the downregulation of cytokines, protecting the endothelium from oxidant injury, and has an essential role in tissue repair [49].

To our knowledge, there is no report about the change of the alveolar number due to cigarette smoke exposure. The number of alveoli in the rat lung ranged from 17.3–24.6 million [51]. CSE in the model rat decreased 47% the number of alveoli, and it may be related to the alveolar cell death. More alveolar cell death followed a collapse of the alveolar, thereby reducing the number of alveoli. CSE in the rat model caused an increase in the percentage of cell death by 5.5-fold than the normal rate, followed by decreases of alveoli number by half of the normal rats. RGFE administration decreased the percentage of cell death followed by increases in the alveoli number. Lycopene can inhibit DNA damage and can localize predominantly within the nuclear membrane to have a protective effect on it [39]. Vitamin C also enhances lung epithelial barrier function by increasing the epigenetic and expression of protein channels at the alveolar-capillary membrane [49]. The pattern of alveolar diameter changes follows MDA levels, alveolar septal thickening, percentage of apoptotic cells, and necrosis. Meanwhile, changes in the number of lung alveolus correspond to changes in cell death and alveolar congestion. It seems there were intercorrelations between these parameters that need to be investigated further.

Toxicity mitigation is important when using herbal ingredients in order to identify its risk on health. Early detection of cellular changes induced by such events could be identified through examination of liver and kidney tissue from rats exposed to chemical insults [52]. The healthy indicator of the liver is serum aspartate aminotransferase, alkaline phosphate, alanine aminotransferase, and bilirubin. Meanwhile, the renal marker is creatinine, urea, uric acid, and blood urea nitrogen [53]. The administration of the *Psidium guajava* fruit extract [54], as well its leaves extract [53], was safe and not toxic to the liver and kidney. Only the repeat administration of *Psidium guajava* bark extract could exhibit mild organ toxicity [55].

CSE causes a dose-dependent upregulation of angiotensin-converting enzyme-2 (ACE2), the SARS-CoV-2 receptor. Chronic smoke exposure triggers the increase in ACE2 expression, which responds to inflammatory signaling and upregulation of viral infections. Smokers are susceptible to SARS-CoV-2 infections and vice versa; SARS-CoV-2 infections could create positive feedback loops that increase ACE2 levels and facilitate viral dissemination [56]. In general, this study revealed that RGFE (specifically, a dose of 5.76 mg/kg BW/day for 44 days) was useful to restore the alveolar cellular damaged by CSE. Therefore, it is necessary the further research the use of RGFE to cure smokers infected with SARS-CoV-2.

## 5. Conclusions

The RGFE has proven to restore the alveolar tissue of rats damaged by exposure to cigarette smoking.

## Data Availability

Data are available from the first author on request.
